# Incorporating biobanking into the future of healthcare: exploring patient and healthcare worker perspectives at a Canadian tertiary academic hospital

**DOI:** 10.1038/s41431-025-01898-7

**Published:** 2025-06-30

**Authors:** Sila Usta, Noor Kundu, Dylan Gowlett-Park, August Lin, Alexandra Misura, Katarina Czibere, Liying Zhang, Olga Bigun, Renato Sasso, Thibika Gunalingam, Winston Ukpong, Tina Khazaee, Betty Wong, Samuel Matsumura, Hubert Tsui, Signy Chow

**Affiliations:** 1 Sunnybrook Biobank, Department of Laboratory Medicine and Molecular Diagnostics, Precision Diagnostics and Therapeutics Program, Sunnybrook Health Sciences Centre, Toronto, ON Canada; 2 Division of Hematological Pathology, Department of Laboratory Medicine and Molecular Diagnostics, Precision Diagnostics and Therapeutics Program, Sunnybrook Health Sciences Centre, Toronto, ON Canada; 3 Sunnybrook Research Institute, University of Toronto, Toronto, ON Canada; 4 Arts and Sciences Cooperative Program, University of Toronto Scarborough, Toronto, ON Canada; 5 Neuroscience Cooperative Program, University of Toronto Scarborough, Toronto, ON Canada; 6 Department of Pathology and Laboratory Medicine, University of Western Ontario, London, ON Canada; 7 Odette Cancer Centre, Sunnybrook Health Sciences Centre, Toronto, ON Canada; 8 Health Sciences Cooperative Program, University of Toronto Scarborough, Toronto, ON Canada; 9 Department of Laboratory Medicine and Pathobiology, Temerty Faculty of Medicine, University of Toronto, Toronto, ON Canada; 10 Department of Immunology, Temerty Faculty of Medicine, University of Toronto, Toronto, ON Canada; 11 Division of Medical Oncology and Hematology, Temerty Faculty of Medicine, University of Toronto, Toronto, ON Canada

**Keywords:** Translational research, Biomarkers, Social sciences

## Abstract

Biobanks are an essential resource for researchers conducting scientific and translational research but require significant support from institutions and healthcare workers (HW) to operate and are reliant on patient consent and participation. In order to better understand the barriers to institution-wide biobanking, we conducted a survey to examine the knowledge, attitudes and concerns of patients and HW on a range of biobanking-related topics, including consenting practices, privacy and trust in the healthcare team and researchers, and current practices at Sunnybrook Health Sciences Centre. Overall, we found that there is strong patient and HW support for biobanking as a resource for research (89–96%). Furthermore, the majority 53% of HW are willing to incorporate biobanking into their clinical workflow and 39% had a neutral response. Encouragingly, patients possess a high level of trust in their healthcare team (80-99%). The main concerns regarding sample donation were ‘breaches of privacy’ and ‘genetic information being used in an exclusionary (discriminatory) fashion.’ Concerns around specimen utilization emerged as a major theme from HW. These results will inform and enhance future biobanking practices to improve the patient experience and increase patient engagement while streamlining specimen collection and utilization for scientific research.

## Background/Introduction

Systematic collection of biological samples is an important resource for scientific research [1]. With a growing demand for personalized medicine, large biobanks are required to accumulate sufficient volumes to study subgroups of illnesses and rare diseases. Utilized effectively, biobanking allows researchers to access a large repository of well-annotated specimens to answer diverse biological and clinical questions. In turn, access to primary tissue facilitates the translation of scientific discoveries to medical practice. Up to 38% of publications in cancer research present data from biospecimens, 31% of which included samples from biorepositories [1].

The practice of biobanking comes with ethical/medico-legal questions related to informed consent, control of samples, incidental findings, and withdrawal of consent [2]. Generally, biobanking practices require a broad consent, as the specific research questions have yet to be defined. Nonetheless, a broad consent still includes some restrictions, such as a field of study or a specific disease, in accordance with guidance by the Canadian Tri-council Policy Statement (TCPS2), and participants may reasonably desire some degree of control over their donated specimens [3]. Research ethical bodies and legislation generally stipulate that informed consent regarding specific risks to personal health information (PHI), including genetic information, must be provided. In Canada, the Canadian Tissue Repository Network (CTRNet), a consortium of tumour banks working to “establish, promote and disseminate standards for biobanking,” provides this framework [4]. Previous research indicates that there is a broad range of public opinions on the topics of consent and privacy, in addition to diversity in standardized practices [5, 6].

The success of biobanking is dependent on the trust and cooperation of participants, and the involvement from multiple disciplines within the healthcare team [7]. Collaborative efforts are required to obtain patient consent in a timely manner and facilitate high-quality biospecimen collection and processing [8]. Structural barriers may include limited personnel and funding resources to support non-clinical work, whereas bedside clinicians may have competing demands on their time, perceived conflicts of interest and competing research studies. Participants may have concerns about donation, including the privacy of their health information and the purpose for which their sample is ultimately used. In spite of this, studies have previously shown that patients are generally supportive of the idea of biobanking, though individual opinions on specific policies vary [5, 7]. Healthcare systems have more recently become prime targets for cyberattacks, and public awareness of the risks to PHI is increasing due to high profile data breaches [9]. Thus, digital privacy challenges may influence potential participants’ willingness to donate samples.

The Sunnybrook Hematology Biobank at Sunnybrook Health Sciences Centre (SHSC) in Toronto, Canada, was established in 2021 to capitalize on the inception of a new acute leukemia referral program. Its mission is to collect and store blood, bone marrow and other biological samples for future research by scientists and clinicians. From 2021-2024, the Sunnybrook Hematology Biobank has collected over 1000 samples from more than 700 patients with acute leukemia, myelodysplastic syndromes, myeloproliferative neoplasms and other hematologic disorders. Interest from other clinicians and investigators led to the establishment of neuroendocrine tumor and central nervous system tumor biobanks in 2023–2024.

The Sunnybrook Hematology Biobank model integrates biobank sample collection at the time of standard-of-care bone marrow procedures. A comprehensive in-person consent discussion by dedicated Biobank staff presents the purpose of the Biobank and reviews the privacy implications of donation. Emphasis is placed on the integration with clinical visits to minimize disruptions in their care.

To further advance an institution-wide biobanking program, we sought to examine present-day attitudes toward biobanking, and evaluate and improve our practices, by conducting a survey of three cohorts: patients who had participated in biobanking (BB), “non-biobank” (NB) patients, and healthcare workers/researchers (HW) at SHSC. The study was approved by the local SHSC Research Ethics Board and supported by a Practice-Based Research and Innovation Seed Grant.

## Methods

### Participants and recruitment

We targeted a total of 300 participants representing 3 cohorts (target *n* = 100 per cohort). Cohort 1: patients who had donated samples to Sunnybrook Biobanks (BB); Cohort 2: patients from the Odette Cancer Centre (OCC) at SHSC who have not participated in biobanking (Non-biobank, NB); and Cohort 3: healthcare workers (HW), inclusive of physicians, nurses, laboratory medicine staff, allied health professionals and administrative staff, and scientists affiliated with SHSC including Sunnybrook Research Institute (SRI). The survey was administered either online using the REDCap platform, or by paper distributed by study staff [10, 11]. BB patients were primarily contacted by telephone and emailed a link to the online survey after agreeing to participate. NB patients were recruited for this study in waiting areas at the OCC and provided paper surveys or a link to the online survey.

HW were primarily recruited by institutional email distribution lists at SHSC and OCC, though paper copies of the survey were also distributed to some laboratory medicine staff. Emails targeted physicians, nurses, allied health professionals, laboratory personnel, and hospital administrators within the Department of Laboratory Medicine and Molecular Diagnostics, the Department of Medicine, the Division of Medical Oncology and Hematology, OCC and scientists within SRI.

### Survey development

Three distinct surveys were generated with identical online (REDcap) and paper formats (Appendix A-C). Survey questions were designed around themes of (1) knowledge and support for biobanking, (2) privacy and control, and (3) trust, based on a systematic review of the literature with additional questions around current institutional practices. BB patient surveys also included questions regarding their experience with the biobanking consenting process and HW surveys contained questions regarding the integration of biobanking into their clinical workflow to assess and improve the quality of local practices at SHSC.

Responses were graded on a 5-point Likert scale: strongly disagree, disagree, neutral, agree, strongly agree [12]. A two-week pilot study was conducted to recruit 8 patients (4 online and 4 paper) and 4 healthcare workers (online) prior to the main study to troubleshoot procedural and technological issues. This data was not used in the final analysis. Recruitment remained open until the target was reached for each cohort.

### Statistical analysis

Paper surveys were input manually to the REDCap application by study staff. Incomplete surveys (<1%) were included in the analysis as each question was analyzed separately. To compare demographics among 3 participant cohorts (NB, BB, HW), Fisher exact test or Chi-square test was performed as appropriate.

For cohort comparisons, an ordinal logistic regression analysis was used to create a cumulative logit prediction model of each Likert scale on the possible predictor of participant cohort (NB, BB, HW). Confounding factors of age and level of education were included in the model. For HW-specific analyses, associations were assessed in relation to professional and research roles. The proportional odds assumption between outcome categories was tested for each ordinal logistic regression model by a Score test. A non-significant Score test indicates that the odds ratios can be interpreted as constant across all possible cut points of the outcome. Pearson χ2 was used to test goodness-of-fit for categorical variables and non-significant Pearson χ2 indicates that an observed frequency distribution did not differ from the predicted distribution. R^2^ was also applied for measure of fit in the modeling and calculated by (L_O_ – L_M_)/L_O_, where L_O_ and L_M_ represent the maximized –2(log likelihood) of the null model and the fitted model, respectively. R^2^ indicates the proportion of variability in the Likert scale which can be explained by the predictive factors. A *p*-value of less than 0.05 was considered statistically significant. All calculations were performed using Statistical Analysis Software (SAS, version 9.4, Cary, NC).

## Results and analysis

### Participants

126 HW, 101 NB, and 100 BB participants completed surveys. The survey was sent to over 2000 HW via institutional email for 126 responses (6.3% response rate). 51% of BB participants that were contacted agreed to participate. The response rate for NB participants was not formally recorded due to the nature of distribution (approaching groups in waiting rooms) but estimated by study staff to be 80-90%.

Of the HW surveyed, 71% were female, the median age group was 35–54 (54%), and the majority identified as White (44%), followed by Chinese (17%). Among NB participants, 63% were female, the majority were aged 55–74 (56%), and 75% identified as White. Of BB participants, 59% were female, the median age group was 55–74 (51%) and 73% identified as White. There were significant differences in age, education, and race/ethnicity amongst the three groups where HW tended to be younger, more educated and more likely to identify as non-white (Table 1). Due to differences between the BB/NB and HW surveys with regards to education, responses were clustered into three groups: vocational, undergraduate and graduate as indicated (Table 1).

**Table 1:** Demographic information for survey respondents in each cohort.

Cohort	Biobank Patients (BB) (*N* = 100)	Non-Biobank Patients (NB) (*N* = 101)	Healthcare Worker (HW) (*N* = 126)	*P*-value
**Gender**				**0.1598**
Female	58.59%	63.27%	70.73%	
Male	41.41%	36.73%	29.27%	
**Age categories**				**<0.0001**
18–34	5.00%	9.28%	20.33%	
35–54	15.00%	21.65%	53.66%	
55–74	51.00%	55.67%	24.39%	
75 or above	29.00%	13.40%	1.63%	
**Education**				**<0.0001**
Undergraduate	29.17%	37.50%	45.45%	
Graduate/Postgraduate	25.00%	29.17%	51.24%	
Technical/Vocational	45.83%	33.33%	3.31%	
**Ethnicity**				
White	73.00%	75.45%	44.19%	**<0.0001**
South Asian	3.00%	0.91%	13.18%	**<0.0001**
Black	6.00%	2.73%	3.10%	
Filipino	3.00%	5.45%	4.65%	
Arab	0.00%	4.55%	1.55%	
Latin American	0.00%	1.82%	2.33%	
Southeast Asian	0.00%	0.00%	1.55%	
West Asian	1.00%	2.73%	2.33%	
Chinese	11.00%	3.64%	17.05%	**<0.0001**
Korean	0.00%	0.00%	1.55%	
Japanese	1.00%	0.00%	0.78%	
Indigenous	2.00%	0.91%	0.00%	
Other group	0.00%	1.82%	0.78%	
Prefer not to answer	0.00%	0.00%	6.98%	

*BB* Biobank Patients, *NB* Non-Biobank Patients, *HW* Healthcare Worker. Differences between cohorts were analyzed using Fischer’s exact test. “Technical/Vocational” includes Medical Laboratory Technician/Technologist (HW survey) and Technical/Vocational (BB/NB survey). “Undergraduate” includes Bachelor of Science, and Registered Nurse (HW survey), and Undergraduate (BB/NB survey). “Postgraduate” includes Master/Doctor of Phil, Medical Doctor (HW survey), graduate degree and postgraduate degree (BB/NB survey).

### Understanding of and support for biobanking

75% of BB participants, 59% of HW and 35% of NB participants believed they had a good understanding of biobanking (agree/strongly agree) with significant differences between cohorts (BB vs NB, *P* < 0.0001; BB vs. HW, *P* = 0.016, Table 2). Higher levels of education were also correlated with a greater understanding of biobanking (Graduate/postgraduate vs undergraduate, *P* = 0.015; Technical/vocational vs undergraduate, *P* = 0.0176). The majority of respondents supported biobanking and agreed/strongly agreed that it is a good strategy for research (96% HW, 89% NB, 96% BB) with BB respondents agreeing more strongly with this statement than NB respondents (*P* = 0.012), and participants aged 55–74 agreeing more strongly than participants aged 18–34 (*P* = 0.012).

The majority of survey participants believed (agree/strongly agree) that more resources should be devoted to creating biobanks (91% BB, 85% NB, 83% HW) and there was a strong desire to integrate this practice into clinical care (93% BB, 91% NB, 90% HW). No differences were observed between participant groups, age or educational attainment groups (Table 2).

**Table 2:** Cohort responses to Likert-scale questions pertaining to the theme of Knowledge and Support for Biobanking.

Cohort	Strongly Agree	Agree	Neutral	Disagree	Strongly Disagree	*P*-value
**I have a good understanding of what biobanking is**						
**HW**	20.63%	38.10%	14.29%	21.43%	5.56%	<0.0001 *BB vs. NB 0.0156 *BB vs. HW
**NB**	6.93%	27.72%	15.84%	22.77%	26.73%	
**BB**	16.16%	58.59%	22.22%	3.03%	0.00%	
**If patients choose to participate, biobanking should be integrated as a part of their routine clinical care, eliminating the need to make additional visits for donation**						
**HW**	42.86%	46.83%	7.94%	2.38%	0.00%	**NS**
**NB**	35.00%	56.00%	6.00%	2.00%	1.00%	
**BB**	45.92%	46.94%	6.12%	0.00%	1.02%	
**More hospitals should devote resources to creating biobanks in order to benefit medical research**						
**HW**	32.54%	50.79%	16.67%	0.00%	0.00%	**NS**
**NB**	34.00%	51.00%	14.00%	0.00%	1.00%	
**BB**	46.46%	44.44%	9.09%	0.00%	0.00%	
**Collecting biological samples and related patient information is an effective strategy for researching disease/cancer**						
**HW**	43.65%	51.59%	3.97%	0.00%	0.79%	0.0118 *BB vs. NB
**NB**	33.00%	56.00%	9.00%	2.00%	0.00%	
**BB**	56.57%	39.39%	3.03%	0.00%	1.01%	

*BB* Biobank Patients, *NB* Non-Biobank Patients, *HW* Healthcare Worker. Ordinal logistic regression analysis was used to create a cumulative logit prediction model using the Likert scale and participant cohort (NB, BB, HW) as possible predictors. *p < 0.05.

### Concerns about biobanking and trust

**Fig. 1:**
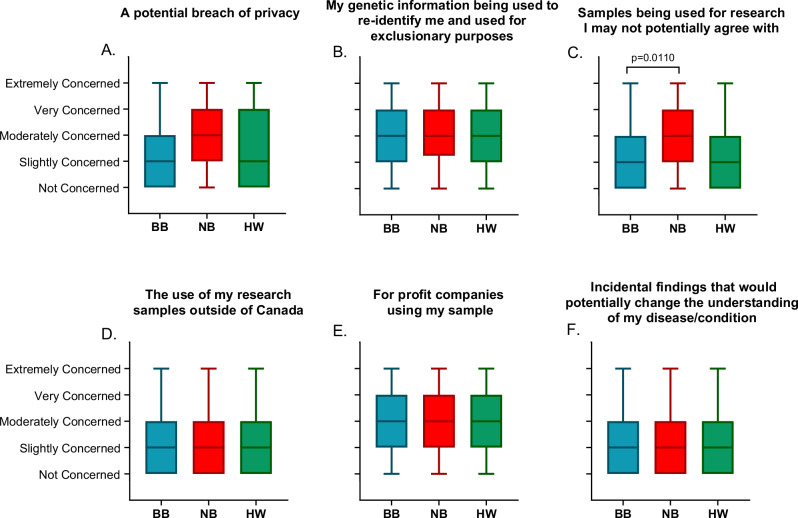
Patient concerns regarding donation of samples for biobanking. **A** A potential breach of privacy. **B** Genetic information being used to re-identify participants and used for exclusionary purposes. **C** Samples being used for research I may not agree with. **D** The use of my research samples outside of Canada. **E** For profit companies using my samples. **F** Incidental findings that would potentially change the understanding of my disease/condition. Whiskers indicate minimum to maximum range, box extends from 25th to 75th percentile. Centre line indicates the median. Ordinal logistic regression analysis was used to create a cumulative logit prediction model using the Likert scale and participant cohort (NB, BB, HW) as possible predictors. *p < 0.05, **p < 0.01, ***p < 0.001, **** *p* = 0.0001.

Top concerns regarding sample donation included concerns over genetic information used to re-identify participants for exclusionary purposes, use of samples by for profit companies, and research that respondents may not agree with. For most concerns, there were no differences between cohorts or differences between age or educational attainment, except that BB participants were less concerned about samples being used for research they did not agree with compared to NB participants (*P* = 0.02) (Fig. 1).

Overall, participants had a high degree of trust (trust/strongly trust) in SHSC physicians (90–99%), nurses (80–91%), laboratory personnel (86–89%), university/hospital research institutions (69–74%, 78–85% respectively) and charitable disease foundations (53–72%). NB and BB participants had a higher degree of trust in Sunnybrook physicians, (*P* = 0.001 and *P* = 0.006), nurses (*P* = 0.0008 and *P* = 0.0076) and charitable disease foundations (*P* = 0.021 and *P* = 0.002) as compared to HW. Irrespective of cohort, higher educational attainment correlated to a higher degree of trust in government research organizations (graduate/postgraduate vs undergraduate, *P* = 0.005), otherwise there were no differences between groups. There were low degrees of trust for for-profit companies (7–14%) and insurance companies (3–9%) among the patients and HW surveyed, with no differences between race/ethnicities (white vs non-white) or education level obtained (Table 3).^1^

**Table 3:** Level of trust in different groups regarding the distribution of participant health information and biological samples.

Indicate your level of trust or distrust in each group regarding the research, handling, and distribution of participant health information and biological samples							
Group	Cohort	Strongly Trust	Trust	Neither Trust/ Distrust	Distrust	Strongly Distrust	P value
**Sunnybrook Physicians**	**HW**	40%	50%	7%	3%	0%	**0.0012** **HW vs. NB **0.0059** **BB vs. HW
	**NB**	57%	37%	4%	1%	1%	
	**BB**	56%	43%	1%	0%	0%	
**Sunnybrook Nurses**	**HW**	31%	49%	14%	5%	1%	**0.0008** ***HW vs. NB **0.0076** **BB vs. HW
	**NB**	50%	40%	8%	1%	1%	
	**BB**	45%	45%	9%	0%	0%	
**Sunnybrook Laboratory Personnel**	**HW**	35%	51%	13%	2%	0%	**NS**
	**NB**	42%	45%	10%	3%	0%	
	**BB**	44%	45%	11%	0%	0%	
**Hospital Research Institutions**	**HW**	30%	55%	13%	2%	1%	**NS**
	**NB**	34%	44%	20%	1%	1%	
	**BB**	35%	51%	14%	0%	0%	
**University research Institutions**	**HW**	21%	53%	22%	2%	2%	**NS**
	**NB**	28%	41%	25%	5%	1%	
	**BB**	24%	49%	25%	1%	0%	
**Government Research Institutions**	**HW**	14%	43%	35%	6%	2%	**NS**
	**NB**	16%	25%	43%	12%	3%	
	**BB**	16%	33%	38%	10%	2%	
**Charitable Disease Based Foundation**	**HW**	10%	44%	40%	4%	2%	**0.0011** **BB vs NB **0.0212** *HW vs. NB **0.0019** **BB vs. HW
	**NB**	17%	36%	34%	10%	3%	
	**BB**	20%	52%	24%	4%	0%	
**For Profit Company Research Institutions**	**HW**	1%	6%	38%	37%	18%	**NS**
	**NB**	6%	8%	38%	36%	11%	
**Insurance companies**	**HW**	0%	3%	21%	36%	40%	**0.0263** *NB vs. HW
	**NB**	3%	6%	27%	40%	24%	

Ordinal logistic regression analysis was used to create a cumulative logit prediction model using the Likert scale and participant cohort (NB, BB, HW) as possible predictors. * *p* < 0.05, **p < 0.01, ***p < 0.001, **** *p* = 0.0001.

### Current consenting practices

78% of BB participants felt that the consent discussion provided them with sufficient understanding regarding biobank participation while 22% responded neutrally. Most participants recalled key elements of the consent discussion including the donation of biological materials (87%), the potential use of their samples for genetic research (66%), and access to their health information related to their disease (64%). Fewer participants recalled elements related to use of their samples after donation (Table 4). 10% of patients did not recall the discussion they had around donating their samples for biobanking, despite selecting the correct BB electronic survey (Table 4).

**Table 4:** Biobank (BB) patients were asked to select which components of the biobank enrolment discussion they recalled.

I recall that the following was discussed during the informed consent discussion:	% of BB
The study involves donating my biological materials (blood, bone marrow, tissue etc.)	87%
The use of my samples may be involved in genetic research	66%
The study involves the use of my health information as related to my disease	64%
My samples may be used by other hospitals and/or universities	48%
I receive no personal benefits from participating in biobanking	48%
Information related to me as it exists in population databases (Statistics Canada, etc.) may be used for research	44%
My sample may be used by for profit drug companies	20%
I do not recall engaging in an informed consent discussion about biobanking	10%

Ordinal logistic regression analysis was used to create a cumulative logit prediction model using the Likert scale and participant cohort (NB, BB, HW) as possible predictors. * *p* < 0.05, **p < 0.01, ***p < 0.001, **** *p* = 0.0001.

Most BB (60%) participants thought that requiring re-consent for specific studies was unnecessary, agreeing/strongly agreeing with the statement “This would be a waste of time and money,” and were more likely to feel this way than NB participants (43%) (*P* < 0.05). NB participants also indicated that they would have more trust and respect for biobank related studies if they were re-approached for consent (31% BB, 50% NB, *P* = 0.002). There was a large proportion of neutral responses towards feeling more in control (48% BB, 43% NB) and more involved in the study (46% BB, 35% NB) with a re-consenting model.

65% of NB and 40% of BB participants felt that they should be able to select which types of research their samples could be used for, with a significant difference between groups (*P* = 0.0002). The majority of participants felt their healthcare experience would be made more meaningful in donating samples for research (79% BB, 83% NB) with no difference between age and educational attainment groups.

### Perceptions amongst healthcare workers (HW)

**Fig. 2:**
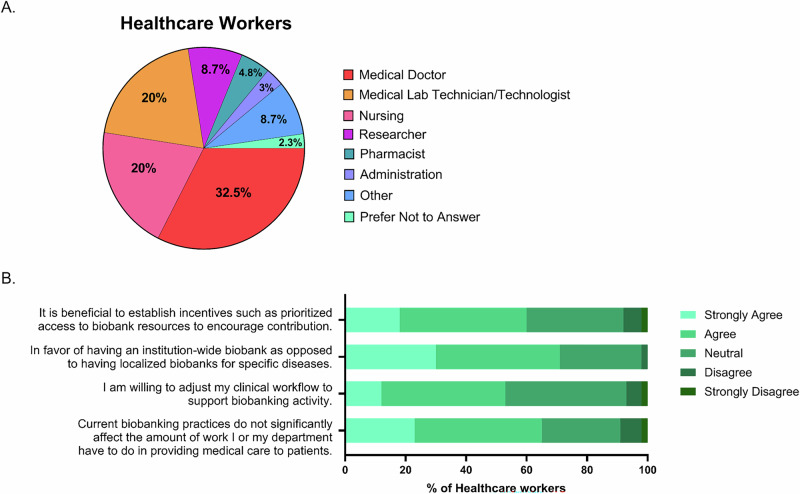
Healthcare worker participants and perspectives. **A** Healthcare worker professions. **B** Healthcare worker perspectives on biobanking practices.

Of the HW respondents, 32.5% were physicians, 20% were nurses, 20% were laboratory technologists/technicians, 8.7% were researchers (Fig. 2A). 45% of HW held academic appointments with a requirement for research (HW-researchers) and 42% would consider accessing the Sunnybrook Biobank or other biobanks for their own research. 40% of respondents had been involved in biobanking practices at Sunnybrook and 65% did not think that current biobanking practices at Sunnybrook had a significant impact on their workload (Fig. 2B). Indeed, 53% of HW were willing to adjust their workflow to support biobanking (agree/strongly agree), including 52% of physicians, 56% of nurses, 48% of Medical Laboratory Technician/Technologists (MLT).

**Fig. 3:**
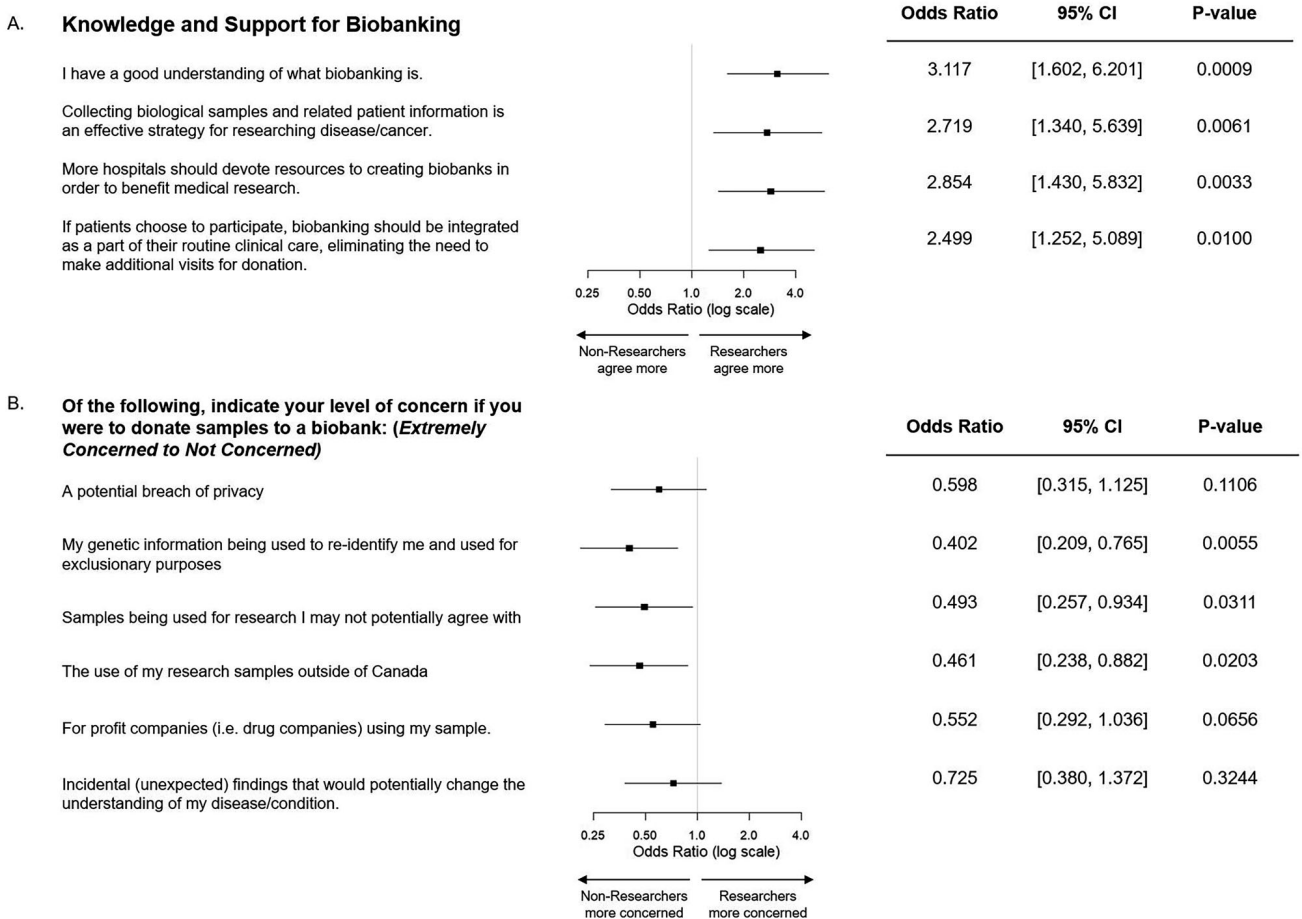
Researcher and Non-Researcher HW Perspectives. **A** Perceptions of biobanking and **B** Potential concerns with biobank donation. Ordinal logistic regression analysis was used to create a cumulative logit prediction model using the Likert scale and healthcare workers with a role in research versus non-researcher healthcare workers as possible predictors. * *p* < 0.05, **p < 0.01, ***p < 0.001, **** *p* = 0.0001.

We compared responses between HW-researchers and HW-non-researchers. HW-researchers had a greater understanding of biobanking (*P* = 0.0009), belief in its efficacy as a research strategy (*P* = 0.0061) and in research incentives (*P* = 0.0028). HW-researchers were also more likely to believe in institutional support for biobanking (*P* = 0.0033) and integration of biobanking with clinical care (*P* = 0.01, Fig. 3A). There were no differences between HW-researchers and HW-non-researchers with regard to perceived impact of current biobanking practices on their workflow or willingness to adjust their clinical workflow to support biobanking.

HW-researchers were also less likely to report concerns with sample donation (Fig. 3B) and had higher rates of trust for Sunnybrook physicians (*P* = 0.0404), laboratory personnel (*P* = 0.0028), government research organizations(*P* = 0.011) and for-profit companies (*P* = 0.0149) compared to HW-non-researchers.

We also compared responses between different HW groups. Overall, there were more differences between nurses compared to researchers and MDs than other comparisons: nurses were less likely to report an understanding of biobanking (*P* = 0.0001), believe that biobanking was a good research strategy (*P* = 0.0004) or believe that hospitals should donate resources to biobanking (*P* = 0.0019) compared to MDs or researchers. They also had more concerns about biobanking compared to MDs or researchers (*P* < 0.05 for 4/6 concerns) (Suppl. Table 1).

From additional submitted comments, an emerging theme amongst HW was concern regarding potential non-use of biobanked samples or the effort/cost to utility ratio. Additional concerns raised included explaining incidental findings to patients, degradation of tissue/samples over time, decreased utility over time, and compromise in clinical testing to prioritize research sample collection. From a donation perspective, concerns included commercialization without compensation, security of personal health information and a desire to know how one’s donated sample(s) contributed to scientific research.

## Discussion

### Support for biobanking

Overall, the majority of respondents supported biobanking and agreed that biobanking practices are important for research, with no differences between age groups or educational level. Unsurprisingly, BB participants who had a consent discussion and donated samples supported this view more strongly than either HW or NB participants. Since it is unlikely that NB participants would have had the opportunity to participate in biobanking, this may reflect increased support for biobanking in those who have experienced the informed consent process rather than a selection bias. Both BB and NB patient cohorts strongly indicated that donating samples to a biobank would foster a sense of meaningful contribution. While motivation for biobanking was not specifically explored, these responses suggest altruistic reasons for donating, in keeping with previous research [7, 13].

There is broad general support for biobanking from HW with 83% agreeing that biobanking is a valuable resource. 52% of HW were willing to provide support to the local biobank with 39% responding neutrally, leaving only 9% not supportive. This is similar to an Australian study, in which 47% of HW responded affirmatively to “I would be willing to help create a Cancer Biobank” and 60% responded affirmatively to “I would be willing to provide a patient information about the Cancer Biobank” [8]. There is widespread agreement among both clinicians and patients that biobanking should be integrated into routine clinical care.

In general, biobanks are financially supported from various sources, most commonly from direct public funds, from the host institution (university/hospital), and indirectly from research grants [14]. Many have mixed models/funding streams. As cost recovery for most biobanking activities is low (as low as 1%), ongoing external funding is required [14]. The majority of HW surveyed here agreed that biobank operations should be sustained by public/institutional funds rather than from individual researchers/grants or donations, and most felt that more resources should be devoted to biobanking. An Australian study has also shown a reduction in public trust in privately funded biobanks relative to publicly funded ones, strengthening the argument for ongoing public support [15]. Further study is warranted to inform decisions around public healthcare, research spending, and resource allocation in the Canadian context.

### Trust in individuals, institutions and research practices

We report a high degree of trust in hospital personnel, researchers, and in university, hospital and government research institutions, particularly from patient participants. In line with previous studies, there were lower degrees of trust in for-profit companies and insurance companies among those surveyed [6, 15]. Retained trust in healthcare teams is reassuring, particularly given broad public perception of declining trust in healthcare professionals in North America, though some studies have shown conflicting results regarding trust in HW during the COVID-19 pandemic [16–18].

Respondents expressed a desire for some level of control over the type of research their samples were being used for, though this did not translate into a preference for a reconsenting/multiple-consent model. BB participants were more likely to indicate that re-consent was unnecessary compared to NB participants. In combination with our findings that most BB patients recalled key components of the consent discussion, these views serve as a positive evaluation of the efficacy and quality of our current consenting practices. We did not find a correlation between preferring a single vs. repeated consent model and educational level obtained observed by Caulfield et al. [19]. We favor a single consent model for practical reasons, with the ability to place restrictions on sample use at the time of consent.

### Concerns with regard to donating samples for biobanking

Top concerns regarding sample donation were (1) genetic information being used to re-identify participants and used for exclusionary (discriminatory) purposes and (2) for-profit companies using the sample. Concerns regarding privacy breaches and discrimination were cited amongst patients that were approached and declined to participate in biobanking, when reasons for declining were provided (internal data).

An emerging theme with both HW and patient participants was a desire for accountability and transparency. In response to “If you have a concern not listed, please specify...”, some HW were concerned that collected samples would remain in storage rather than used for research. BB participants also voiced the desire to know that their samples were being used as intended (internal communications). Sample under-utilization is a key issue in the biobanking community, with some studies reporting <10% utilization rate [20]. Indefinite storage, compounded by underutilization, also impacts environmental and financial sustainability, of which there is also growing awareness [21]. Thus, ongoing biobanking practices must be cognizant of the utilization metrics to gauge sustainability and continue to receive support from key stakeholders.

### Healthcare researchers

HW-researchers have dual responsibilities with both patient care and research priorities. While a significant difference between HW-researchers and HW-non-researchers was found in their respective understanding and concerns about biobanking, this did not impact the willingness to adjust clinical workflows to facilitate sample collection. Differences amongst healthcare professions also influenced concerns related to biobanking, with the biggest differences between nurses and researchers. However, this may simply reflect differences between HW-researchers and HW-non-researchers since the majority of nurses surveyed here have clinical roles without research requirements whereas physicians were split between researchers and non-researchers (Supplementary Table 1). Educational initiatives may be warranted to address the lower rates of reported understanding of biobanking in non-researchers and nurses relative to other groups.

42% of HW expressed a desire to access biobanked material for their own research, similar to the Australian group, in which 57% reported they “would be likely to use information from the Cancer biobank in future.” A majority of HW (58%) reported that incentives to access biobanked material/information would be helpful to promote biobanking. Academic healthcare centres with combined research and healthcare mandates are poised to drive large-scale, integrated biobanking practices that could support clinician-researchers and affiliated scientists in their research endeavors. Our survey responses support such a model.

### Current consenting and biobanking practices

Current BB participants reported high levels of comfort with the consent discussion, though a 22% neutral response to “I have a good understanding of what biobanking is.” indicates room for improvement. It is reassuring that a high proportion of patients (88%) recalled discussing the donation of their biologic materials and potential use for genetic research (67%). However, 10% of patients who had donated samples do not recall engaging in a discussion about biobanking and only half recalled discussions around the use of their samples outside of the institution. Reasons for this cannot be ascertained from this study, however, possible explanations may be (1) biobanking consent often coincided with a new cancer diagnosis and associated emotional stress (2) participants may not retain specific details due to the period of time elapsed since consenting (some were surveyed up to 3 years after the original donation).

Our current model for the Sunnybrook Hematology Biobank integrates biobank sample collection at the time of standard-of-care bone marrow procedure. This model allows the clinician to obtain verbal consent for specimen collection in urgent clinical situations followed thereafter by a full informed consent discussion by biobank staff regarding the implications of sample donation to reduce disruption to the clinical workflow. Our high consent rate (90% in 2024, internal data), and a high proportion of positive to neutral responses (62% agree/strongly agree, 25% neutral) suggest that our current practices do not significantly impact clinical workload.

The consent discussion begins with an explanation biobanking and its purpose. Emphasis is placed on aligning sample with clinical timepoints, eliminating the need for additional visits, and collecting samples through the same venipuncture (blood), aspiration needle (bone marrow), or operation (surgical specimen). Our protocol also allows for biobanking of clinical surplus, where remnant blood/marrow specimens are rerouted from the clinical/flow cytometry laboratory after clinical processing. Patients may additionally limit the access to their samples (i.e. decline access to industry or out-of-institution/out-of-country researchers). Patients are also given the opportunity to ask questions about biobanking and the donation process. We feel that the extent of our discussion, the integration with clinical workflow and the opportunity to ask questions are essential elements of the process that contribute to our success.

## Limitations and conclusions

As is common with voluntary surveys, our study is limited by a selection bias, including bias toward women, English speakers, younger and more able populations, including those without hearing or reading difficulties. The survey did not specifically include patients that had been asked but declined to participate in biobanking, which may have led to additional insights.

Despite limitations, we are confident in the overall results demonstrating a high degree of support for the current practices of biobanking at our institution, including high level of trust from the local healthcare team. Alongside the reported desire by both patient participants and healthcare workers to integrate biobanking into clinical practice, we identified an appeal for greater transparency and accountability as to the actual use of collected samples. These results will inform future improvements to our consenting model and integration with clinical practices. The call to provide up-to-date reporting of the research being conducted with biobanked samples is an encouraging sign of proactive engagement with research initiatives from both healthcare workers and the general public. Additional studies and ongoing discussion with patient participants and healthcare/research teams is needed around the issues of resource allocation and funding support for biobanks.

## Supplementary information

Biobank Survey for Biobank (BB) Patient Participants

Biobank Survey for Non-Biobank (NB) Patient Participants

Biobank Survey for Healthcare Workers (HW)

Comparison of Opinions on Biobanking in Different Healthcare Workers

## Data Availability

Survey files are included in this study as supplementary files. Datasets generated during this analysis are available from the corresponding author on request.

## Footnotes

1 BB participants were not given questions regarding for-profit companies and insurance companies
