# Supplementary material for: Incorporating biobanking into the future of healthcare: exploring patient and healthcare worker perspectives at a Canadian tertiary academic hospital
Source: Eur J Hum Genet. 2025 Jun 30;33(9):1194–202. doi: 10.1038/s41431-025-01898-7 (PMC12402111; doi:10.1038/s41431-025-01898-7)
Supplement: Supplementary file 1 — Biobank Survey for Biobank (BB) Patient Participants [file 41431_2025_1898_MOESM1_ESM.pdf]

Appendix A – Biobank Patient Participant Survey

Exploring Patient Perspectives on Biobanking

Principal Investigator:
Samuel Matsumura
Ph#416-480-6100, ext. 685231
samuel.matsumura@sunnybrook.ca

Research Ethics Board Chairman:
Dr. Brian Murray
Ph#416-480-6100, ext. 62461
brian.murray@sunnybrook.ca

Sponsor: Practice Based Research and Innovation Seed Grant Program

PLEASE READ THE FOLLOWING TEXT BEFORE COMPLETING THIS SURVEY

You are being asked to participate in a survey. We hope to better understand patient and health care worker perspectives on biobanking (the collection of biological specimens for future research). This survey will help to guide the future of biobanking at Sunnybrook. This survey consists of 17 questions; we anticipate it will take 15 minutes or less. More information can be found at the end of the survey on page 10.

To receive your compensation ($5 gift card to Tim Hortons), we may ask for your name and/or email. This will not be linked to your responses.

Name:_______________________________________

Email:_______________________________________

Questionnaire

Section A: Demographic Information

Section A contains demographic information such as age and gender which will be collected for analysis. You can choose NOT to answer these questions and skip to section B if you prefer. Please read through each question carefully before answering.

1. Please select your gender:

- ○ Female
- ○ Male
- ○ Other (please specify)____
- ○ Prefer not to answer

2. Please select your age range:

- ○ 18-24
- ○ 25-34
- ○ 35-44
- ○ 45-54
- ○ 55-64
- ○ 65-74
- ○ 75-84
- ○ 85 years old or above
- ○ Prefer not to answer

3. Please select your ethnicity (select all that apply):

☐ White

☐ South Asian (e.g., East Indian, Pakistani, Sri Lankan)

☐ Black

☐ Filipino

☐ Arab

☐ Latin American

☐ Southeast Asian (e.g., Vietnamese, Cambodian, Laotian, Thai)

☐ West Asian (e.g., Iranian, Afghan)

☐ Chinese

☐ Korean

☐ Japanese

☐ Indigenous

☐ Other group (*Please specify*):_____________________________________

○ Prefer not to answer

4. Highest level of education obtained (select the most relevant):

○ Some secondary (high school) education

○ High school diploma or equivalent (e.g., GED)

○ Technical and/or vocational certificate/diploma

○ Undergraduate degree (e.g., Bachelor of Science, Bachelor of Art)

○ Graduate degree (e.g., Master of Science, Master of Art)

○ Post graduate degree (e.g., Doctor of Philosophy, Doctor of Medicine)

○ None of the above

○ Prefer not to answer

Section B: Knowledge and Support for Biobanking

The rest of this survey will contain statements and questions asking your opinion on biobanking. Please read each one carefully and select to what extent you agree or disagree with the statements/questions. Alternatively, select the most representative answer to the statements/questions.

5. Have you agreed to donate biological material (blood, tissue, etc.) to a biobank at Sunnybrook (e.g., Tissue Bank, Hematology Biobank, Brain Biobank)?

○ Yes

○ No

○ Unsure

6. I recall that the following was discussed during the informed consent discussion:

☐ The study involves donating my biological materials (blood, bone marrow, tissue etc.)

☐ The study involves the use of my health information as related to my disease

☐ The use of my samples may be involved in genetic research

☐ Information related to me as it exists in population databases (smoker's data, etc.) may be used for research

☐ My samples may be used by other hospitals and/or universities

☐ My sample may be used by for profit drug companies

☐ I receive no personal benefits from participating in biobanking

☐ I do not recall engaging in an informed consent discussion about biobanking

7. The consent discussion I had provided me with a complete understanding of my role and involvement in the study.

Strongly Disagree	Disagree	Neutral	Agree	Strongly Agree
○	○	○	○	○

8. Collecting biological samples and related patient information is an effective strategy for researching disease/cancer.

Strongly Disagree	Disagree	Neutral	Agree	Strongly Agree
○	○	○	○	○

9. More hospitals should devote resources to creating biobanks in order to benefit medical research (e.g. collected biological samples and/or distributed disease related information to researchers).

Strongly Disagree	Disagree	Neutral	Agree	Strongly Agree
○	○	○	○	○

10. If patients choose to participate, biobanking should be integrated as a part of their routine clinical care, eliminating the need to make additional visits for donation.

Strongly Disagree	Disagree	Neutral	Agree	Strongly Agree
○	○	○	○	○

11. I have a good understanding of what biobanking is.

Strongly Disagree	Disagree	Neutral	Agree	Strongly Agree
○	○	○	○	○

12. Of the following (continues onto next page), indicate your level of concern when donating samples to a biobank.

a) A potential breach of privacy (i.e. unauthorized personnel accessing patient information).

Not concerned	Slightly concerned	Moderately concerned	Very concerned	Extremely concerned
○	○	○	○	○

b) My genetic information being used to re-identify me and used for exclusionary purposes (e.g. being denied insurance due to my genetic profile).

Not concerned	Slightly concerned	Moderately concerned	Very concerned	Extremely concerned
○	○	○	○	○

c) Samples being used for research I may not potentially agree with (i.e. animal testing, genetic testing).

Not concerned	Slightly concerned	Moderately concerned	Very concerned	Extremely concerned
○	○	○	○	○

d) The potential use of my research samples outside of Canada.

Not concerned	Slightly concerned	Moderately concerned	Very concerned	Extremely concerned
○	○	○	○	○

e) For profit companies (i.e. drug companies) potentially using my sample.

Not concerned	Slightly concerned	Moderately concerned	Very concerned	Extremely concerned
○	○	○	○	○

f) Incidental (unexpected) findings that would potentially change the understanding of my disease/condition.

Not concerned	Slightly concerned	Moderately concerned	Very concerned	Extremely concerned
○	○	○	○	○

If you have a concern not listed above, please specify here:

____

Section C: Patient Experience

13. How would you feel if you had to give permission repeatedly to use your stored biological sample and health information before each new research study?

a) I would feel that it was a waste of time and money.

Strongly Disagree	Disagree	Neutral	Agree	Strongly Agree
○	○	○	○	○

b) I would feel bothered (continues onto next page).

Strongly Disagree	Disagree	Neutral	Agree	Strongly Agree
○	○	○	○	○

c) I would feel I have control.

Strongly Disagree	Disagree	Neutral	Agree	Strongly Agree
○	○	○	○	○

d) I would have more trust in the study.

Strongly Disagree	Disagree	Neutral	Agree	Strongly Agree
○	○	○	○	○

e) I would feel respected and involved.

Strongly Disagree	Disagree	Neutral	Agree	Strongly Agree
○	○	○	○	○

14. My overall healthcare experience has been or would be more meaningful knowing that my donated samples may be used for research.

Strongly Disagree	Disagree	Neutral	Agree	Strongly Agree
○	○	○	○	○

15. Patients should be able to select which specific types of research their donated samples can be used for (e.g. genetic research, animal testing, using population databases, etc.)

Strongly Disagree	Disagree	Neutral	Agree	Strongly Agree
○	○	○	○	○

16. If research samples unexpectedly reveal information that might be important for a patient's medical care, who should be informed about these results? (*Select all that apply)*

☐ Patient/Family

☐ Treating doctor/specialist at the hospital where the samples were biobanked

☐ Family doctor

☐ None of the above

☐ Other (Please specify)_______________________________________

Section D: Trust

17. Indicate your level of trust or distrust in each group regarding the research, handling, and distribution of participant health information and biological samples (continue to next page to complete)

	**Strongly Distrust**	**Distrust**	**Neither Trust nor Distrust**	**Trust**	**Strongly Trust**
**Sunnybrook Physicians**	○	○	○	○	○
**Sunnybrook Nurses**	○	○	○	○	○
**Sunnybrook Laboratory Personnel**	○	○	○	○	○
**Hospital Research Institutions** (e.g. researchers, scientists)	○	○	○	○	○

	Strongly Distrust	Distrust	Neither Trust nor Distrust	Trust	Strongly Trust
**University Research Institutions**	○	○	○	○	○
**Government Research Institutions** (e.g. Stats Canada)	○	○	○	○	○
**Charitable Disease Based Foundation** (e.g. Leukemia and Lymphoma Society of Canada)	○	○	○	○	○

Thank you for completing our survey. Please hand the survey back to the Research Associate at your earliest convenience to receive your compensation.

Feel free to reach out to us at sunnybrook.biobank@sunnybrook.ca or call us at (416) 480-6100 Extension: 88162

More Information

You do not need to have participated in biobanking to take this survey. The study's duration will last 2 years. You may take as much time as you wish to decide whether or not to participate. Please ask the study staff or one of the investigator(s) to clarify anything you do not understand. You are NOT obligated to complete this survey and can exit this survey at any time. Your participation in this survey does not waive any of your legal rights. We do not anticipate there will be any physical risks to participating in this study. In the unlikely event that you are injured due to the study team's actions or negligence, compensation will be provided as required by law and regulations. The Sunnybrook Biobank team will use reasonable measures within its control to safeguard your information.
