# Supplementary material for: Incorporating biobanking into the future of healthcare: exploring patient and healthcare worker perspectives at a Canadian tertiary academic hospital
Source: Eur J Hum Genet. 2025 Jun 30;33(9):1194–202. doi: 10.1038/s41431-025-01898-7 (PMC12402111; doi:10.1038/s41431-025-01898-7)
Supplement: Supplementary file 3 — Biobank Survey for Healthcare Workers (HW) [file 41431_2025_1898_MOESM3_ESM.pdf]

Appendix C - Health Worker Survey

Exploring Health Worker perspectives on Biobanking

Principal Investigator:
Samuel Matsumura
Ph#416-480-6100, ext. 685231
samuel.matsumura@sunnybrook.ca

Research Ethics Board Chairman:
Dr. Brian Murray
Ph#416-480-6100, ext. 62461
brian.murray@sunnybrook.ca

Sponsor: Practice Based Research and Innovation Seed Grant Program

PLEASE READ THE FOLLOWING TEXT BEFORE COMPLETING THIS SURVEY

You are being asked to participate in a survey. We hope to better understand patient and health care worker perspectives on biobanking (the collection of biological specimens for future research). This survey will help to guide the future of biobanking at Sunnybrook. This survey consists of 21 questions; we anticipate it will take 15 minutes or less. More information can be found at the end of the survey on page 10.

Questionnaire

Section A: Demographic Information

Section A contains demographic information such as age and gender which will be collected for analysis. You can choose NOT to answer these questions and skip to section B if you prefer. Please read through each question carefully before answering.

1. Please select your gender:

○ Female

○ Male

○ Other (please specify)____

○ Prefer not to answer

2. Please select your age range:

○ 18-24

○ 25-34

○ 35-44

○ 45-54

○ 55-64

○ 65-74

○ 75-84

○ 85 years old or above

○ prefer not to answer

3. Please select your ethnicity (select all that apply):

- ☐ White
- ☐ South Asian (e.g., East Indian, Pakistani, Sri Lankan)
- ☐ Black
- ☐ Filipino
- ☐ Arab
- ☐ Latin American
- ☐ Southeast Asian (e.g., Vietnamese, Cambodian, Laotian, Thai)
- ☐ West Asian (e.g., Iranian, Afghan)
- ☐ Chinese
- ☐ Korean
- ☐ Japanese
- ☐ Indigenous
- ☐ Other (*Please specify):*____
- ○ Prefer not to answer

4. What is your professional role?

- ○ Medical Doctor
- ○ Medical Lab Technician/Technologist
- ○ Medical Student
- ○ Pharmacist
- ○ Nurse Practitioner
- ○ Researcher
- ○ Registered Nurse
- ○ Nursing Student
- ○ Prefer not to answer
- ○ Other (*Please specify):*____

5. Highest level of education obtained (check all that apply):

- ☐ Bachelor of Science
- ☐ Master of Science/ Master of Science in Nursing
- ☐ Doctor of Philosophy/ Doctor of Nursing Practice
- ☐ Medical Doctor
- ☐ Registered Nurse
- ☐ Medical Lab Technician
- ○ None of the above
- ○ Prefer not to answer

6. Does your academic job description include a requirement for research activity?

- ○ Yes
- ○ No
- ○ Not applicable

7. Would you consider accessing the Sunnybrook Biobank or other biobanks for your own research?

- ○ Yes
- ○ No
- ○ Not applicable

8. What is your Program/ Departmental affiliation with Sunnybrook *(Please specify below)*:

____

Section B: Knowledge and Support for Biobanking

The rest of this survey will contain several statements and questions asking your opinion on biobanking. Please read each one carefully and select to what extent you agree or disagree with the statements/questions. Alternatively, select the most representative answers to the statements/questions.

9. I am currently, or have been, actively involved with biobanking practices at Sunnybrook (i.e. biobank specimen collection, introducing biobanking to patients and/or related practices etc.)

○ Yes

○ No

○ Unsure

10. I have a good understanding of what biobanking is.

Strongly Disagree	Disagree	Neutral	Agree	Strongly Agree
○	○	○	○	○

11. Collecting biological samples and related patient information is an effective strategy for researching disease/cancer.

Strongly Disagree	Disagree	Neutral	Agree	Strongly Agree
○	○	○	○	○

12. More hospitals should devote resources to creating biobanks in order to benefit medical research (e.g. collected biological samples and/or distributed disease related information to researchers).

Strongly Disagree	Disagree	Neutral	Agree	Strongly Agree
○	○	○	○	○

13. If patients choose to participate, biobanking should be integrated as a part of their routine clinical care, eliminating the need to make additional visits for donation.

Strongly Disagree	Disagree	Neutral	Agree	Strongly Agree
○	○	○	○	○

14. Of the following, indicate your level of concern if you were to donate samples to a biobank.

a) A potential breach of privacy (i.e. unauthorized personnel accessing patient information).

Not concerned	Slightly concerned	Moderately concerned	Very concerned	Extremely concerned
○	○	○	○	○

b) My genetic information being used to re-identify me and used for exclusionary purposes (e.g. being denied insurance due to my genetic profile).

Not concerned	Slightly concerned	Moderately concerned	Very concerned	Extremely concerned
○	○	○	○	○

c) Samples being used for research I may not potentially agree with (i.e. animal testing, genetic testing).

Not concerned	Slightly concerned	Moderately concerned	Very concerned	Extremely concerned
○	○	○	○	○

d) The use of my research samples outside of Canada.

Not concerned	Slightly concerned	Moderately concerned	Very concerned	Extremely concerned
○	○	○	○	○

e) For profit companies (i.e. drug companies) using my sample.

Not concerned	Slightly concerned	Moderately concerned	Very concerned	Extremely concerned
○	○	○	○	○

f) Incidental (unexpected) findings that would potentially change the understanding of my disease/condition.

Not concerned	Slightly concerned	Moderately concerned	Very concerned	Extremely concerned
○	○	○	○	○

If you have a concern not listed above, please specify below:

____

Section C: Biobanking and Health Workers

15. Current biobanking practices do not significantly affect the amount of work I or my department have to do in providing medical care to patients.

Strongly Disagree	Disagree	Neutral	Agree	Strongly Agree
○	○	○	○	○

16. I am willing to adjust my clinical workflow to support biobanking activity.

Strongly Disagree	Disagree	Neutral	Agree	Strongly Agree
○	○	○	○	○

17. I am in favor of having an institution-wide biobank as opposed to having localized biobanks for specific diseases.

Strongly Disagree	Disagree	Neutral	Agree	Strongly Agree
○	○	○	○	○

18. To encourage healthcare professionals to contribute to biobanking efforts, it is beneficial to establish incentives such as prioritized access to biobank resources, financial incentives, and opportunities for authorship in related papers.

Strongly Disagree	Disagree	Neutral	Agree	Strongly Agree
○	○	○	○	○

19. I am aware that biobanking is a strategic-direction-1 priority at Sunnybrook Health Sciences Centre.

○ Yes

○ No

○ Unsure

20. Biobanking operations should be sustained by… (rank in order of priority from 1-5 and only use each number once; 1- highest, 5-lowest):

__ The Sunnybrook Health Sciences Centre

__ Individual disease site groups interested in biobanking (i.e. hematologists, oncologists, surgeons)

__ Independent investigators wishing to use the biobank

__ Direct donations or Sunnybrook Foundation

__ The Sunnybrook Research Institute

Section D: Trust

21. Indicate your level of trust or distrust in each group regarding the research, handling, and distribution of participant health information and biological samples (continue onto next page to complete).

	Strongly Distrust	Distrust	Neither Trust nor Distrust	Trust	Strongly Trust
**Sunnybrook Physicians**	○	○	○	○	○
**Sunnybrook Nurses**	○	○	○	○	○
**Sunnybrook Laboratory Personnel**	○	○	○	○	○
**Hospital Research Institutions** (e.g. researchers, scientists)	○	○	○	○	○
**University Research Institutions**	○	○	○	○	○
**Government Research Institutions** (e.g. Stats Canada)	○	○	○	○	○
**Charitable Disease Based Foundation** (e.g. Leukemia and Lymphoma Society of Canada)	○	○	○	○	○

	Strongly Distrust	Distrust	Neither Trust nor Distrust	Trust	Strongly Trust
**For Profit Company Research Institutions** (e.g. drug companies)	○	○	○	○	○
**Insurance companies**	○	○	○	○	○

Thank you for completing our survey. Please hand the survey back to the Research Associate at your earliest convenience.

Feel free to reach out to us at sunnybrook.biobank@sunnybrook.ca or call us at (416) 480-6100 Extension: 88162.

More Information

You do not need to have participated in biobanking to take this survey. The study's duration will last 2 years. You may take as much time as you wish to decide whether or not to participate. Please ask the study staff or one of the investigator(s) to clarify anything you do not understand.

You are NOT obligated to complete this survey and can exit this survey at any time. Your participation in this survey does not waive any of your legal rights. We do not anticipate there will be any physical risks to participating in this study.

In the unlikely event that you are injured due to the study team's actions or negligence, compensation will be provided as required by law and regulations. The Sunnybrook Biobank team will use reasonable measures within its control to safeguard your information.
