# Supplementary material for: Incorporating biobanking into the future of healthcare: exploring patient and healthcare worker perspectives at a Canadian tertiary academic hospital
Source: Eur J Hum Genet. 2025 Jun 30;33(9):1194–202. doi: 10.1038/s41431-025-01898-7 (PMC12402111; doi:10.1038/s41431-025-01898-7)
Supplement: Supplementary file 4 — Comparison of Opinions on Biobanking in Different Healthcare Workers [file 41431_2025_1898_MOESM4_ESM.docx]

|  |  | **Parameter Estimation** | | **Model Fitting Information** | | |
| --- | --- | --- | --- | --- | --- | --- |
| **Outcome** | **Independent Variable** | **p-value** | **OR (95% CI)** | **Proportional Odds assumption p-value** | **Pearson χ2 statistic  (p-value)** | **R^2^** |
| Q9 (Yes vs. No/Unsure) | Type of HCW (overall effect) | 0.2650 |  | NA | NA | 3.90% |
|  | MD vs. MLTT | 0.1719 | 2.250 (0.813, 6.599) |  |  |  |
|  | Nurse vs. MLTT | 0.2368 | 2.250 (0.719, 7.378) |  |  |  |
|  | Researcher vs. MLTT | 0.3071 | 0.900 (0.199, 3.663) |  |  |  |
|  | MD vs. Nurse | 0.9999 | 1.000 (0.363, 2.751) |  |  |  |
|  | MD vs. Researcher | 0.1721 | 2.499 (0.671, 9.305) |  |  |  |
|  | Nurse vs. Researcher | 0.2026 | 2.499 (0.611, 10.223) |  |  |  |
| ***Likert Scale (Strongly Agree to Strongly Disagree)*** | | | | | | |
| **q10** | **Type of HCW (overall effect)** | **0.0001** |  | **0.5135** | **8.81 (0.4550)** | **19.92 %** |
|  | MD vs. MLTT | 0.1573 | 3.416 (1.344, 8.930) | . |  |  |
|  | **Nurse vs. MLTT** | **0.0009** | **0.745 (0.281, 1.964)** | **.** |  |  |
|  | **Researcher vs. MLTT** | **0.0004** | **10.613 (2.966, 41.442)** | **.** |  |  |
|  | **MD vs. Nurse** | **0.0016** | **4.582 (1.780, 11.798)** | **.** |  |  |
|  | MD vs. Researcher | 0.0602 | 0.322 (0.099, 1.050) | . |  |  |
|  | **Nurse vs. Researcher** | **<.0001** | **0.070 (0.019, 0.263)** | **.** |  |  |
| **q11** | **Type of HCW (overall effect)** | **0.0004** |  | **0.1020** | **10.61 (0.1012)** | **19.83 %** |
|  | MD vs. MLTT | 0.3621 | 2.861 (1.070, 7.978) | . |  |  |
|  | **Nurse vs. MLTT** | **0.0006** | **0.501 (0.151, 1.570)** | **.** |  |  |
|  | **Researcher vs. MLTT** | **0.0020** | **13.486 (2.931, 98.827)** | **.** |  |  |
|  | **MD vs. Nurse** | **0.0019** | **5.714 (1.897, 17.214)** | **.** |  |  |
|  | MD vs. Researcher | 0.0606 | 0.212 (0.042, 1.071) | . |  |  |
|  | **Nurse vs. Researcher** | **0.0003** | **0.037 (0.006, 0.216)** | **.** |  |  |
| **q12** | **Type of HCW (overall effect)** | **0.0019** |  | **0.0686** | **7.50 (0.0575)** | **23.41 %** |
|  | MD vs. MLTT | 0.1265 | 1.159 (0.452, 2.987) | . |  |  |
|  | **Nurse vs. MLTT** | **0.0004** | **0.455 (0.155, 1.305)** | **.** |  |  |
|  | **Researcher vs. MLTT** | **0.0005** | **33.139 (5.495, 643.670)** | **.** |  |  |
|  | MD vs. Nurse | 0.0659 | 2.545 (0.940, 6.885) | . |  |  |
|  | **MD vs. Researcher** | **0.0020** | **0.035 (0.004, 0.294)** | **.** |  |  |
|  | **Nurse vs. Researcher** | **0.0001** | **0.014 (0.001, 0.126)** | **.** |  |  |
| **q13** | **Type of HCW (overall effect)** | **0.0426** |  | **0.1283** | **8.00 (0.2383)** | **7.69 %** |
|  | MD vs. MLTT | 0.1377 | 2.342 (0.905, 6.223) | . |  |  |
|  | **Nurse vs. MLTT** | **0.0329** | **0.702 (0.238, 2.038)** | **.** |  |  |
|  | Researcher vs. MLTT | 0.1054 | 3.029 (0.809, 12.373) | . |  |  |
|  | **MD vs. Nurse** | **0.0184** | **3.338 (1.225, 9.098)** | **.** |  |  |
|  | MD vs. Researcher | 0.6785 | 0.773 (0.229, 2.611) | . |  |  |
|  | **Nurse vs. Researcher** | **0.0310** | **0.232 (0.061, 0.875)** | **.** |  |  |
| ***Likert Scale (Extremely Concerned to Not Concerned)*** | | | | | | |
| **Q14a** | **Type of HCW (overall effect)** | **0.0453** |  | **0.5485** | **8.27 (0.5068)** | **7.58 %** |
|  | MD vs. MLTT | 0.1300 | 0.428 (0.172, 1.048) | . |  |  |
|  | Nurse vs. MLTT | 0.0515 | 1.202 (0.454, 3.200) | . |  |  |
|  | Researcher vs. MLTT | 0.1065 | 0.344 (0.107, 1.080) | . |  |  |
|  | **MD vs. Nurse** | **0.0248** | **0.356 (0.144, 0.877)** | **.** |  |  |
|  | MD vs. Researcher | 0.6957 | 1.244 (0.416, 3.723) | . |  |  |
|  | **Nurse vs. Researcher** | **0.0397** | **3.496 (1.061, 11.519)** | **.** |  |  |
| **Q14b** | **Type of HCW (overall effect)** | **0.0022** |  | **0.0884** | **14.00 (0.1224)** | **13.63 %** |
|  | MD vs. MLTT | 0.0861 | 0.262 (0.102, 0.653) | . |  |  |
|  | Nurse vs. MLTT | 0.0505 | 0.784 (0.300, 2.035) | . |  |  |
|  | **Researcher vs. MLTT** | **0.0112** | **0.153 (0.044, 0.505)** | **.** |  |  |
|  | **MD vs. Nurse** | **0.0178** | **0.335 (0.135, 0.827)** | **.** |  |  |
|  | MD vs. Researcher | 0.3345 | 1.720 (0.572, 5.172) | . |  |  |
|  | **Nurse vs. Researcher** | **0.0080** | **5.141 (1.532, 17.247)** | **.** |  |  |
| **Q14c** | **Type of HCW (overall effect)** | **0.0002** |  | **<.0001** | **10.73 (0.2946)** | **19.63 %** |
|  | MD vs. MLTT | 0.5706 | 0.884 (0.360, 2.165) | . |  |  |
|  | **Nurse vs. MLTT** | **<.0001** | **2.958 (1.096, 8.179)** | **.** |  |  |
|  | **Researcher vs. MLTT** | **0.0001** | **0.122 (0.028, 0.454)** | **.** |  |  |
|  | **MD vs. Nurse** | **0.0104** | **0.299 (0.119, 0.753)** | **.** |  |  |
|  | **MD vs. Researcher** | **0.0027** | **7.261 (1.988, 26.528)** | **.** |  |  |
|  | **Nurse vs. Researcher** | **<.0001** | **24.306 (5.896, 100.205)** | **.** |  |  |
| **Q14d** | **Type of HCW (overall effect)** | **0.0039** |  | **<.0001** | **17.28 (0.0446)** | **13.09 %** |
|  | MD vs. MLTT | 0.9413 | 0.865 (0.341, 2.203) | . |  |  |
|  | **Nurse vs. MLTT** | **0.0005** | **2.825 (1.010, 8.096)** | **.** |  |  |
|  | **Researcher vs. MLTT** | **0.0063** | **0.249 (0.057, 0.940)** | **.** |  |  |
|  | **MD vs. Nurse** | **0.0113** | **0.306 (0.122, 0.765)** | **.** |  |  |
|  | MD vs. Researcher | 0.0549 | 3.470 (0.974, 12.360) | . |  |  |
|  | **Nurse vs. Researcher** | **0.0005** | **11.336 (2.884, 44.553)** | **.** |  |  |
| Q14e | Type of HCW (overall effect) | 0.1068 |  | 0.5987 | 6.56 (0.6830) | 5.73 % |
|  | MD vs. MLTT | 0.9428 | 0.834 (0.337, 2.056) | . |  |  |
|  | Nurse vs. MLTT | 0.0457 | 1.542 (0.569, 4.208) | . |  |  |
|  | Researcher vs. MLTT | 0.0301 | 0.348 (0.102, 1.156) | . |  |  |
|  | MD vs. Nurse | 0.1755 | 0.541 (0.222, 1.316) | . |  |  |
|  | MD vs. Researcher | 0.1183 | 2.398 (0.800, 7.189) | . |  |  |
|  | Nurse vs. Researcher | 0.0147 | 4.436 (1.340, 14.692) | . |  |  |
| Q14f | Type of HCW (overall effect) | 0.0904 |  | 0.1385 | 11.99 (0.2141) | 6.38 % |
|  | MD vs. MLTT | 0.8118 | 1.223 (0.493, 3.074) | . |  |  |
|  | Nurse vs. MLTT | 0.0150 | 2.535 (0.936, 7.019) | . |  |  |
|  | Researcher vs. MLTT | 0.0796 | 0.554 (0.152, 1.898) | . |  |  |
|  | MD vs. Nurse | 0.1150 | 0.483 (0.195, 1.194) | . |  |  |
|  | MD vs. Researcher | 0.1773 | 2.206 (0.699, 6.967) | . |  |  |
|  | Nurse vs. Researcher | 0.0170 | 4.572 (1.312, 15.936) | . |  |  |
| ***Likert Scale (Strongly Agree to Strongly Disagree)*** | | | | | | |
| q15 | Type of HCW (overall effect) | 0.9487 |  | 0.7314 | 5.87 (0.7529) | 0.37 % |
|  | MD vs. MLTT | 0.7215 | 0.918 (0.359, 2.338) | . |  |  |
|  | Nurse vs. MLTT | 0.6024 | 1.211 (0.439, 3.354) | . |  |  |
|  | Researcher vs. MLTT | 0.9095 | 0.966 (0.257, 3.639) | . |  |  |
|  | MD vs. Nurse | 0.5538 | 0.758 (0.303, 1.897) | . |  |  |
|  | MD vs. Researcher | 0.9372 | 0.950 (0.267, 3.383) | . |  |  |
|  | Nurse vs. Researcher | 0.7428 | 1.254 (0.325, 4.834) | . |  |  |
| q16 | Type of HCW (overall effect) | 0.4064 |  | 0.5215 | 8.44 (0.4909) | 2.80 % |
|  | MD vs. MLTT | 0.5277 | 1.244 (0.494, 3.156) | . |  |  |
|  | Nurse vs. MLTT | 0.5704 | 1.238 (0.458, 3.363) | . |  |  |
|  | Researcher vs. MLTT | 0.0998 | 3.274 (0.822, 13.578) | . |  |  |
|  | MD vs. Nurse | 0.9909 | 1.005 (0.399, 2.533) | . |  |  |
|  | MD vs. Researcher | 0.1467 | 0.380 (0.103, 1.404) | . |  |  |
|  | Nurse vs. Researcher | 0.1682 | 0.378 (0.095, 1.508) | . |  |  |
| q17 | Type of HCW (overall effect) | 0.6168 |  | 0.7572 | 3.22 (0.7804) | 1.69 % |
|  | MD vs. MLTT | 0.2581 | 1.243 (0.499, 3.104) | . |  |  |
|  | Nurse vs. MLTT | 0.3998 | 0.684 (0.246, 1.883) | . |  |  |
|  | Researcher vs. MLTT | 0.7078 | 0.775 (0.226, 2.641) | . |  |  |
|  | MD vs. Nurse | 0.2085 | 1.816 (0.717, 4.605) | . |  |  |
|  | MD vs. Researcher | 0.4133 | 1.603 (0.518, 4.962) | . |  |  |
|  | Nurse vs. Researcher | 0.8399 | 0.882 (0.262, 2.970) | . |  |  |
| **q18** | **Type of HCW (overall effect)** | **0.0320** |  | **0.0001** | **17.17 (0.0461)** | **9.09 %** |
|  | MD vs. MLTT | 0.6792 | 0.933 (0.362, 2.399) | . |  |  |
|  | **Nurse vs. MLTT** | **0.0031** | **0.301 (0.108, 0.816)** | **.** |  |  |
|  | Researcher vs. MLTT | 0.0999 | 1.666 (0.490, 5.767) | . |  |  |
|  | **MD vs. Nurse** | **0.0198** | **3.093 (1.196, 7.997)** | **.** |  |  |
|  | MD vs. Researcher | 0.3364 | 0.560 (0.171, 1.827) | . |  |  |
|  | **Nurse vs. Researcher** | **0.0096** | **0.181 (0.050, 0.659)** | **.** |  |  |
| Q19 (Yes vs. No/Unsure) | Type of HCW (overall effect) | 0.0797 |  | NA | NA | 7.10% |
|  | MD vs. MLTT | 0.1489 | 1.841 (0.663, 5.398) |  |  |  |
|  | Nurse vs. MLTT | 0.0278 | 0.429 (0.100, 1.599) |  |  |  |
|  | Researcher vs. MLTT | 0.1326 | 2.250 (0.591, 8.859) |  |  |  |
|  | MD vs. Nurse | 0.0210 | 4.295 (1.246, 14.805) |  |  |  |
|  | MD vs. Researcher | 0.7469 | 0.818 (0.242, 2.768) |  |  |  |
|  | Nurse vs. Researcher | 0.0299 | 0.191 (0.043, 0.851) |  |  |  |
| ***Likert Scale (Highest to Lowest)*** | | | | | | |
| Q20a | Type of HCW (overall effect) | 0.2215 |  | 0.0002 | 10.75 (0.2932) | 5.02 % |
|  | MD vs. MLTT | 0.1882 | 0.941 (0.370, 2.388) | . |  |  |
|  | Nurse vs. MLTT | 0.4142 | 1.037 (0.357, 3.025) | . |  |  |
|  | Researcher vs. MLTT | 0.0391 | 4.130 (0.958, 22.196) | . |  |  |
|  | MD vs. Nurse | 0.8533 | 0.907 (0.324, 2.541) | . |  |  |
|  | MD vs. Researcher | 0.0407 | 0.228 (0.055, 0.940) | . |  |  |
|  | Nurse vs. Researcher | 0.0794 | 0.251 (0.054, 1.176) | . |  |  |
| Q20b | Type of HCW (overall effect) | 0.0952 |  | 0.1814 | 10.74 (0.2939) | 6.20 % |
|  | MD vs. MLTT | 0.4819 | 0.973 (0.395, 2.394) | . |  |  |
|  | Nurse vs. MLTT | 0.0548 | 1.545 (0.553, 4.368) | . |  |  |
|  | Researcher vs. MLTT | 0.0173 | 0.258 (0.064, 1.018) | . |  |  |
|  | MD vs. Nurse | 0.3453 | 0.630 (0.241, 1.645) | . |  |  |
|  | MD vs. Researcher | 0.0444 | 3.770 (1.034, 13.743) | . |  |  |
|  | Nurse vs. Researcher | 0.0121 | 5.985 (1.480, 24.204) | . |  |  |
| Q20c | Type of HCW (overall effect) | 0.1070 |  | <.0001 | 17.48 (0.0418) | 6.39 % |
|  | MD vs. MLTT | 0.0139 | 2.080 (0.851, 5.184) | . |  |  |
|  | Nurse vs. MLTT | 0.6194 | 0.833 (0.270, 2.560) | . |  |  |
|  | Researcher vs. MLTT | 0.2367 | 0.569 (0.137, 2.197) | . |  |  |
|  | MD vs. Nurse | 0.0687 | 2.496 (0.932, 6.684) | . |  |  |
|  | MD vs. Researcher | 0.0516 | 3.657 (0.991, 13.493) | . |  |  |
|  | Nurse vs. Researcher | 0.5969 | 1.465 (0.356, 6.028) | . |  |  |
| Q20d | Type of HCW (overall effect) | 0.3698 |  | 0.0669 | 12.60 (0.1816) | 3.56 % |
|  | MD vs. MLTT | 0.5321 | 2.076 (0.815, 5.402) | . |  |  |
|  | Nurse vs. MLTT | 0.3175 | 2.455 (0.852, 7.225) | . |  |  |
|  | Researcher vs. MLTT | 0.9560 | 1.775 (0.525, 6.015) | . |  |  |
|  | MD vs. Nurse | 0.7334 | 0.846 (0.322, 2.219) | . |  |  |
|  | MD vs. Researcher | 0.7909 | 1.170 (0.367, 3.734) | . |  |  |
|  | Nurse vs. Researcher | 0.6189 | 1.384 (0.385, 4.972) | . |  |  |
| **Q20e** | **Type of HCW (overall effect)** | **0.0335** |  | **0.0141** | **16.28 (0.0612)** | **9.14 %** |
|  | **MD vs. MLTT** | **0.0497** | **0.267 (0.097, 0.705)** | **.** |  |  |
|  | Nurse vs. MLTT | 0.1263 | 0.283 (0.098, 0.794) | . |  |  |
|  | Researcher vs. MLTT | 0.4391 | 0.658 (0.195, 2.224) | . |  |  |
|  | MD vs. Nurse | 0.8973 | 0.941 (0.375, 2.365) | . |  |  |
|  | MD vs. Researcher | 0.1386 | 0.406 (0.123, 1.339) | . |  |  |
|  | Nurse vs. Researcher | 0.1967 | 0.431 (0.120, 1.547) | . |  |  |
| ***Likert Scale (Strongly Trust to Strongly Distrust)*** | | | | | | |
| **Q21a** | **Type of HCW (overall effect)** | **0.0279** |  | **<.0001** | **7.34 (0.2904)** | **8.66 %** |
|  | MD vs. MLTT | 0.1879 | 2.558 (0.975, 6.906) | . |  |  |
|  | **Nurse vs. MLTT** | **0.0379** | **0.830 (0.284, 2.412)** | **.** |  |  |
|  | **Researcher vs. MLTT** | **0.0497** | **4.103 (1.112, 16.718)** | **.** |  |  |
|  | **MD vs. Nurse** | **0.0259** | **3.081 (1.145, 8.289)** | **.** |  |  |
|  | MD vs. Researcher | 0.4515 | 0.623 (0.182, 2.133) | . |  |  |
|  | **Nurse vs. Researcher** | **0.0193** | **0.202 (0.053, 0.772)** | **.** |  |  |
| Q21b | Type of HCW (overall effect) | 0.1786 |  | 0.2020 | 8.41 (0.2096) | 4.52 % |
|  | MD vs. MLTT | 0.3919 | 2.571 (1.005, 6.730) | . |  |  |
|  | Nurse vs. MLTT | 0.9710 | 1.985 (0.721, 5.564) | . |  |  |
|  | Researcher vs. MLTT | 0.2611 | 3.192 (0.900, 11.851) | . |  |  |
|  | MD vs. Nurse | 0.5890 | 1.295 (0.507, 3.311) | . |  |  |
|  | MD vs. Researcher | 0.7130 | 0.805 (0.254, 2.550) | . |  |  |
|  | Nurse vs. Researcher | 0.4535 | 0.622 (0.180, 2.154) | . |  |  |
| **Q21c** | **Type of HCW (overall effect)** | **0.0125** |  | **0.0643** | **8.14 (0.2282)** | **10.12 %** |
|  | MD vs. MLTT | 0.2043 | 1.811 (0.710, 4.684) | . |  |  |
|  | **Nurse vs. MLTT** | **0.0032** | **0.441 (0.150, 1.263)** | **.** |  |  |
|  | **Researcher vs. MLTT** | **0.0451** | **2.979 (0.808, 12.027)** | **.** |  |  |
|  | **MD vs. Nurse** | **0.0050** | **4.111 (1.531, 11.039)** | **.** |  |  |
|  | MD vs. Researcher | 0.4203 | 0.608 (0.181, 2.040) | . |  |  |
|  | **Nurse vs. Researcher** | **0.0049** | **0.148 (0.039, 0.560)** | **.** |  |  |
| Q21d | Type of HCW (overall effect) | 0.3058 |  | 0.9338 | 4.02 (0.9103) | 3.43 % |
|  | MD vs. MLTT | 0.4412 | 1.889 (0.715, 5.094) | . |  |  |
|  | Nurse vs. MLTT | 0.2834 | 1.044 (0.358, 3.044) | . |  |  |
|  | Researcher vs. MLTT | 0.1892 | 2.611 (0.737, 9.528) | . |  |  |
|  | MD vs. Nurse | 0.2300 | 1.810 (0.687, 4.770) | . |  |  |
|  | MD vs. Researcher | 0.5874 | 0.723 (0.225, 2.329) | . |  |  |
|  | Nurse vs. Researcher | 0.1583 | 0.400 (0.112, 1.429) | . |  |  |
| Q21e | Type of HCW (overall effect) | 0.3718 |  | 0.4660 | 10.14 (0.3392) | 3.13 % |
|  | MD vs. MLTT | 0.2663 | 1.656 (0.635, 4.375) | . |  |  |
|  | Nurse vs. MLTT | 0.1750 | 0.752 (0.262, 2.143) | . |  |  |
|  | Researcher vs. MLTT | 0.4587 | 1.638 (0.440, 6.174) | . |  |  |
|  | MD vs. Nurse | 0.1088 | 2.203 (0.839, 5.786) | . |  |  |
|  | MD vs. Researcher | 0.9857 | 1.011 (0.308, 3.314) | . |  |  |
|  | Nurse vs. Researcher | 0.2363 | 0.459 (0.126, 1.666) | . |  |  |
| **Q21f** | **Type of HCW (overall effect)** | **0.0197** |  | **<.0001** | **9.64 (0.3805)** | **9.40 %** |
|  | MD vs. MLTT | 0.2148 | 1.726 (0.679, 4.439) | . |  |  |
|  | **Nurse vs. MLTT** | **0.0049** | **0.463 (0.159, 1.314)** | **.** |  |  |
|  | Researcher vs. MLTT | 0.0539 | 2.680 (0.793, 9.263) | . |  |  |
|  | **MD vs. Nurse** | **0.0074** | **3.729 (1.423, 9.769)** | **.** |  |  |
|  | MD vs. Researcher | 0.4508 | 0.644 (0.205, 2.021) | . |  |  |
|  | **Nurse vs. Researcher** | **0.0067** | **0.173 (0.049, 0.615)** | **.** |  |  |
| **Q21g** | **Type of HCW (overall effect)** | **0.0307** |  | **0.0009** | **19.41 (0.0219)** | **8.06 %** |
|  | **MD vs. MLTT** | **0.0292** | **3.825 (1.458, 10.400)** | **.** |  |  |
|  | Nurse vs. MLTT | 0.2007 | 3.089 (1.086, 9.062) | . |  |  |
|  | Researcher vs. MLTT | 0.3563 | 1.378 (0.382, 5.052) | . |  |  |
|  | MD vs. Nurse | 0.6592 | 1.238 (0.479, 3.205) | . |  |  |
|  | MD vs. Researcher | 0.0849 | 2.776 (0.869, 8.868) | . |  |  |
|  | Nurse vs. Researcher | 0.2037 | 2.241 (0.646, 7.781) | . |  |  |
| Q21h | Type of HCW (overall effect) | 0.0819 |  | 0.6292 | 5.73 (0.7663) | 6.32 % |
|  | MD vs. MLTT | 0.6895 | 2.025 (0.806, 5.170) | . |  |  |
|  | Nurse vs. MLTT | 0.1871 | 1.171 (0.409, 3.363) | . |  |  |
|  | Researcher vs. MLTT | 0.0291 | 4.502 (1.289, 16.503) | . |  |  |
|  | MD vs. Nurse | 0.2461 | 1.730 (0.685, 4.365) | . |  |  |
|  | MD vs. Researcher | 0.1763 | 0.450 (0.141, 1.432) | . |  |  |
|  | Nurse vs. Researcher | 0.0356 | 0.260 (0.074, 0.913) | . |  |  |
| **Q21i** | **Type of HCW (overall effect)** | **0.0096** |  | **0.3893** | **5.32 (0.5030)** | **11.18 %** |
|  | **MD vs. MLTT** | **0.0026** | **0.410 (0.156, 1.060)** | **.** |  |  |
|  | Nurse vs. MLTT | 0.5242 | 0.811 (0.285, 2.293) | . |  |  |
|  | **Researcher vs. MLTT** | **0.0079** | **2.992 (0.897, 10.364)** | **.** |  |  |
|  | MD vs. Nurse | 0.1541 | 0.505 (0.198, 1.292) | . |  |  |
|  | **MD vs. Researcher** | **0.0010** | **0.137 (0.042, 0.447)** | **.** |  |  |
|  | **Nurse vs. Researcher** | **0.0386** | **0.271 (0.079, 0.934)** | **.** |  |  |

Supplementary Table 1: Comparison of Opinions on Biobanking in Different Healthcare Workers. Question numbers relate to the Healthcare Worker Survey in Supplementary Figure 3.
